# Nanostructured MOS Sensor for the Detection, Follow up, and Threshold Pursuing of *Campylobacter Jejuni* Development in Milk Samples

**DOI:** 10.3390/s20072009

**Published:** 2020-04-03

**Authors:** Estefanía Núñez-Carmona, Marco Abbatangelo, Dario Zappa, Elisabetta Comini, Giorgio Sberveglieri, Veronica Sberveglieri

**Affiliations:** 1 Institute of Bioscience and Bioresources, CNR-IBBR, 50019 Sesto Fiorentino (FI), Italy; veronica.sberveglieri@ibbr.cnr.it; 2 Department of Information Engineering, University of Brescia, 25123 Brescia (BS), Italy; m.abbatangelo@unibs.it (M.A.); dario.zappa@unibs.it (D.Z.); elisabetta.comini@unibs.it (E.C.); giorgio.sberveglieri@unibs.it (G.S.); 3 NANO SENSOR SYSTEMS, Dep. Information Engineering, NASYS spin-off University of Brescia, 25123 Brescia (BS), Italy

**Keywords:** MOS sensor, nanowires, milk, *Campylobacter* spp., GC-MS

## Abstract

Food poisoning is still the first cause of hospitalization worldwide and the most common microbial agent, *Campylobacter jejuni*, is the most commonly reported gastrointestinal disease in humans in the EU (European Union) as is reported by the European Union One Health 2018 Zoonoses Report styled by the EFSA (European Food Safety Authority) and ECDC (European Center for Disease Prevention and Control). One of the vehicles of transmission of this disease is milk. Nanostructured MOS (Metal Oxide Semiconductor) sensors have extensively demonstrated their ability to reveal the presence and follow the development of microbial species. The main objective of this work was to find a set up for the detection and development follow up of *C. jejuni* in milk samples. The work was structured in two different studies, the first one was a feasibility survey and the second one was to follow up the development of the bacteria inside milk samples. The obtained results of the first study demonstrate the ability of the sensor array to differentiate the contaminated samples from the control ones. Thanks to the second study, it has been possible to find the limit of microbial safety of the contaminated milk samples.

## 1. Introduction

Organizations worldwide run and rule all aspects of the food safety, security, and trading at a global level. Organizations such as the EFSA (European Food Safety Agency), WHO (World Health Organization), and FAO (Food and Agriculture Organization) guarantee the most safe food industry environment we have ever enjoyed, putting the risk perception regarding the food industry at the bottom of the concerning list of those in non-developing countries [1,2]. Even with all the measurements applied to reach food safety worldwide, food poisoning is still the first cause of hospitalization globally. The CDC (Center of Disease Control) estimates that each year, 48 million people get sick from a foodborne illness, 128,000 are hospitalized, and 3000 die just in the USA alone [3].

The European Union One Health 2018 Zoonoses Report styled by the EFSA and ECDC (European Food Safety Authority and European Centre for Disease Prevention and Control) approved 19 November, 2019 encloses a list of the Zoonoses included in compulsory annual monitoring (Directive 2003/99/EC List A) [4].

This report claims that Campylobacteriosis is the most commonly reported gastrointestinal disease in humans in the EU and has been so since 2005. The most common sources for the FBOs (food borne outbreaks) were milk and broiler meat, as in previous years [4,5]. The etiologic vector of the illness is *Campylobacter jejuni*, which is associated with warm-blooded animals involving livestock animals that have shown the microorganisms in their feces [6,7]. Some strains of *C. jejuni* produce a thermolabile enterotoxin (CJT), [8,9] that affects the gastrointestinal system.

*Campylobacter* spp. infection can also be transmitted through unpasteurized milk ingestion when a cow has *C. jejuni* cells in its udder or when milk is contaminated with manure. *Campylobacter jejuni* is a common raw milk contaminant and is notoriously difficult to isolate from food products, because of its fastidious growth requirements [10,11,12,13,14].

Recently, different kinds of sensor have been used for the detection of *C. jejuni*. Some involve the use of DNA detection or oligonucleotide hybridization with the specific target of the pathogen using optical [15], acoustic [16], and electrochemical [17,18] techniques. In other cases, quartz crystal microbalance (QCM) hybridized immunosensors using monoclonal and polyclonal antibodies have been used [19]. Normally DNA or immune based techniques are costly and require a high level expertise to be applied. In recent research, two-dimensional nanostructured materials have been widely used for gas sensing [20]. Furthermore, previous research [21] have used MOS nanostructured gas sensors for the detection of the aforementioned pathogen using lab standard conditions. The main differences regarding the volatile compounds present in the head space between the contaminated samples and the controls ones (performed as sterile liquid culture media) reside in the presence of a higher concentration in alcohol compounds in *C. jejuni* samples than the control one. This increased concentration of alcohols, together with the neoformation of pyrazines, comes as a result of sugar fermentation.

Due to the proven possibility of *C. jejuni* detection using MOS gas sensors, a rapid, easy to use, and economic alarm system that provides enough and accurate information could be constructed in order to actuate corrective and timely actions in order to prevent contamination and spread of the pathogen in the dairy industry. In this work, we illustrate how through the use of nanostructured MOS sensors it is possible to detect, follow up, and conduct threshold detection pursuing of *Campylobacter jejuni* development in milk samples with a fast and economic approach. The use of this kind of technology have been largely applied to obtain remarkable results in the agri food field [22,23,24,25,26].

## 2. Materials and Methods

All the research described in this paper was performed in two different sets of analysis including different techniques. The first one corresponds to a feasibility study of the detection of the pathogen in milk samples, the second one was dedicated to reducing the initial concentration of the microbial agent in the challenge test in order to define the threshold detection of the S3.

### 2.1. Samples Preparation

Starter cultures were prepared in the same way for the two sets of analysis following the same conditions used in previous work [21] with some modifications. *Campylobacter jejuni* subsp. *jejuni* type strain cells were purchased from DMSZ (The Leibniz Institute DSMZ—German Collection of Microorganisms and Cell Cultures GmbH-Deutsche Sammlung von Mikroorganismen und Zellkulturen-), DSM number 4688, (ATCC 33560, CCUG 11284, CIP 702, NCTC 11351). Microbial cells were inoculated in tubes containing 9 mL of Brain Heart Infusion Broth (BHI) liquid media purchased from Sigma Aldrich (Merck). Tubes were incubated in aerobic conditions for 24 h at 35 °C. After the incubation, the culture was used to inoculate tubes of sterile BHI media in order to perform the subsequent dilution. 

For the first set of analysis, the same optical density (OD) as of the number 3 standard of McFarland that corresponds to a concentration of 9 × 10^8^ CFU (Colony Forming Unit) by mL [27] was reached. Afterward, sterile BHI media tubes were used to perform serial dilutions in order to decrease two orders of magnitude reaching 9 × 10^6^ CFU/mL. Subsequently sterile chromatography vials of 20 mL containing 4 mL of sterile whole milk were inoculated with 100 μL using the previous solution (9 × 10^6^ CFU/mL), which leads to a final concentration in the vial near 2.20 × 10^5^ CFU/mL. Control samples were performed using 20 mL sterile chromatographic vials containing 4 mL of sterile whole milk.

Regarding the second set of analysis, the samples were performed in the same way as the previous, but reducing the microbial loading in the final samples by an order of magnitude, which leads to a final concentration in a vial of 2.20 × 10^4^ CFU/mL. Control samples were performed exactly in the same way as the first set of analysis. For this set, the analysis time was performed in just 7 h, so a total number of nine samples was prepared from which two were control samples.

### 2.2. Microbial Analysis

Plate count technique has been applied in every step of the different analysis for the first set of analysis, so every 21 h of analysis from 0 h to 93 h in order to verify, first the number of inoculated cells and second, how the microorganisms will develop inside the sample. The used media were BHI agar plates that once inoculated were incubated at 35 °C for 48 h before counting. Regarding the second set of analysis of measurement, plate count was applied at 0 h and at the end of the analysis after 7 h following the same conditions.

### 2.3. S3 Analysis

In order to operate the system, the S3 instrument was coupled with the autosampler headspace system HT2010H (HTA s.r.l., Brescia, Italy). This autosampler is equipped with a thermostatic shaking oven to equilibrate the sample headspace and a 42-loading position carrousel. The S3 device and the sensor arrays used in this work were constructed and designed by the SENSOR Laboratory (University of Brescia, Italy) in collaboration with NASYS S.r.l., a spin-off of the University of Brescia. This device has demonstrated remarkable performances as has been broadly reported in the literature [28,29,30,31]. S3 device components can be divided in three different groups:

- Pneumatic components: that moves the gas sample to the sensing chamber.
- Electronic boards: that are in charge of data acquisition and transmission to the dedicated WebApp and at the same time allows the synchronization between S3 and the HT2010H.
- Sensing chamber in which up to 10 MOX sensor could be located and is thermally isolated from the surrounding environment.

The sensing array is composed of different sensing elements based on metal oxide semiconducting (MOS) materials. Each sensor consists of a MOS sensing layer deposited on an insulating alumina substrate. The sensing principle is the variation of the electrical conductance of the sensing material upon interaction with the surrounding atmosphere. Target molecules react with adsorbed oxygen species on sensing the material surface, releasing electrons that modulate the electrical properties including the electrical conductance. 

**Figure 1:**
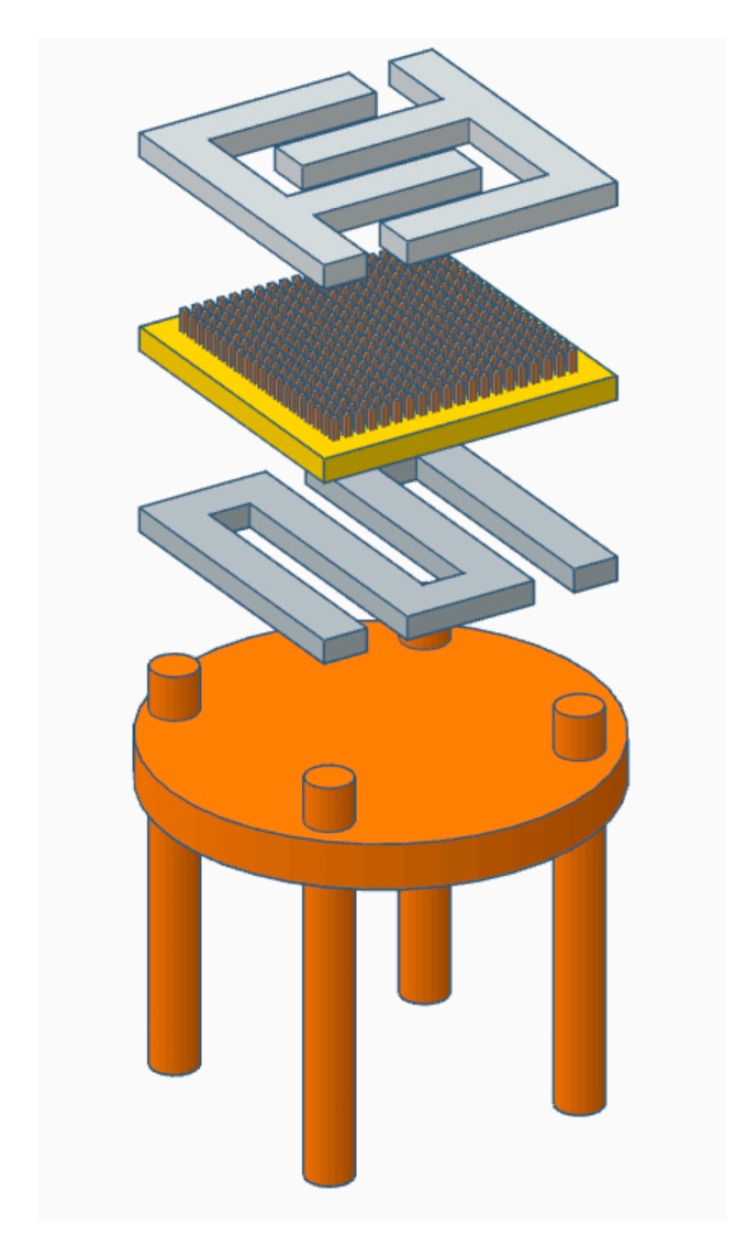
Scheme of the sensor architecture. From top to the bottom: two interdigited platinum electrodes (gray), the sensing material (dark gray), alumina substrate (yellow), platinum heather (gray), gold-coated “transistor outline” (TO-39) package (brown).

For this purpose, two interdigited platinum electrodes were deposited by sputtering technique on top of the sensing material. The kinetic of the chemical reaction was thermally promoted. Therefore, on the backside of the substrate, we deposited a platinum heater to precisely control the working temperature of each device. Finally, each substrate was mounted on a gold-coated “transistor outline” (TO-39) package by electrosoldered gold wires (Figure 1).

To perform the base line of the sensors, the surrounding atmosphere was filtered using an aluminum cylinder of 21.5 cm in length and 5 cm in width, filled with active carbons. Two different arrays were used for this analysis, one for the first set and the other for the second set of analysis using similar sensors in order to find the best performing combination.

To construct the sensor array of the S3 device, six MOX gas sensors were selected for each step of the analysis. Both used arrays were a mix between tin oxide nanowire [32,33] sensors grown by means of the vapor liquid solid technique [34], and the other sensors were prepared with RGTO (Rheotaxial Growth and Thermal Oxidation) thin film technology [35]. The sensor array of the first and second set of analysis are described and represented in Table 1, which shows the material, the sensor type, the functionalization (if this is the case), and the operating temperature. Since the sensors are aspecific, the selection of sensors with different materials and architecture were selected in order to cover all the volatile finger prints of the different samples. For each sensor, the response (AR/R) to 5 ppm of ethanol, selectivity (response ethanol/response carbon monoxide), and limit of detection (LOD) of ethanol for both S3 sensors arrays have been represented (Table 1).

The analysis carried out by the S3 system for each sample can be divided in different steps for a total analysis time of 30 min by vial:

- 5 min of sample gas in contact with the sensors array.
- 5 min to clean the actuators (tubes, pump, sensing chamber, etc.) of the gas sample.
- 20 min to restore the base line.

The sample arrangements for the two different sets of analysis was different. Regarding the first one (feasibility study), analysis was carried out from 0 to 93 h with a total number of 168 samples that were analyzed continuously. Since the used auto sampler has 42 loading positions, four steps of 21 hours were performed in sequence in order to have continuous control of the changes in the head space of the samples. To the extent of also having a continuous survey of the control samples, seven of the aforementioned samples were positioned inside every carrousel in order to perform them every 3 h at a rate of 5 (150 min) contaminated samples to one control (30 min).

In the second set (define the threshold), the total analysis time was performed in only one step, of 7 h of analysis, with a total number of samples of nine. Control samples were analyzed every 3 h with a rate of five to one as well as in the first set of analysis.

In previous work, *C. jejuni* was detected in standard laboratory conditions using the culture media as background for the detection of *C. jejuni* volatiloma [21]. Since the sensors are aspecific and the ultimate goal is to obtain an array that is able to detect the presence of the pathogen in real conditions, new training with real samples (milk) became mandatory to train the sensor array.

### 2.4. S3 Data Analysis

Al the sensors responses were recorded in terms of resistance (Ω) and at the same time normalized to the first value of the acquisition (R0) using MATLAB® R2015a software (MathWorks, USA). Regarding the feature extraction, ΔR/R0 was used for the data analysis. It was performed calculating the difference between the first value and the minimum value for all sensors. Once the data matrix was calculated, principal component analysis (PCA) was applied to the data in order to verify the increment of the CFU/mL in relationship with the variation of the VOCs (Volatile Organic Compound).

## 3. Results and Discussion

In the first set of analysis, the theoretical initial concentration of microbial cell inoculation in whole milk at T0 was 2.20 × 10^5^ CFU/mL. Count plate technique confirmed the real number of inoculated cells was 2.92 × 10^5^ CFU/mL at T0, while the control samples where completely clean throughout the entire analysis time of the first set of analysis. The conversely inoculated sample exhibited an increase in the cell number during the analysis time of the first set of analysis, arriving at 2.19 × 10^9^ CFU/mL, enhancing the initial concentration of nearly four orders of magnitude.

**Figure 2:**
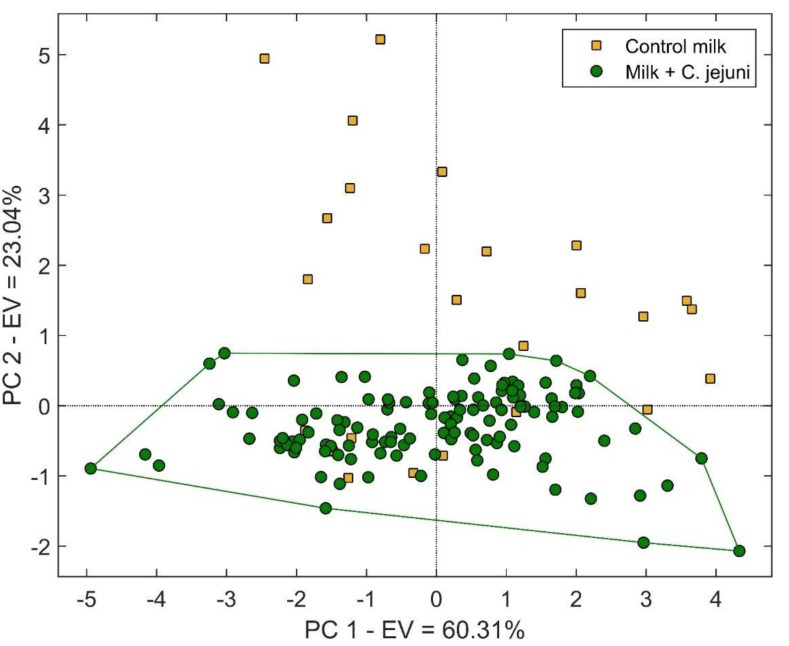
Principle component analysis (PCA) score plot representing the data obtained during the 93 h of the first set of analysis where contaminated samples are dots colored in green while control samples are yellow squares. The horizontal axis represents the explained variance enclosed in PC1 as equal to 60.31%, conversely, the vertical axis represents the explained variance enclosed in PC2 as equal to 23.04%. The total explained variance enclosed in the graphic is equal to 83.35%.

Moreover, regarding the results obtained with the S3 device in the first set of analysis, data are represented in the form of PCA in Figure 2.

A clear separation of two clusters is evident in Figure 1, diversifying contaminated samples out of contaminated ones. Apart from these data, no evolution of the contaminated samples was evident which formed a cluster with no differentiation between the less contaminated and the ones that exhibited a big contamination at the end of the 93 h. This output is mainly due to a saturation phenomenon of the sensor response. 

In the second set of analysis, a reduction of one order of magnitude was performed in order to find out if it was possible to follow up the microbial development of *C. jejuni* inside whole milk samples. The results obtained with the plate count technique were in accordance with those obtained with the sensor device S3. 

**Figure 3:**
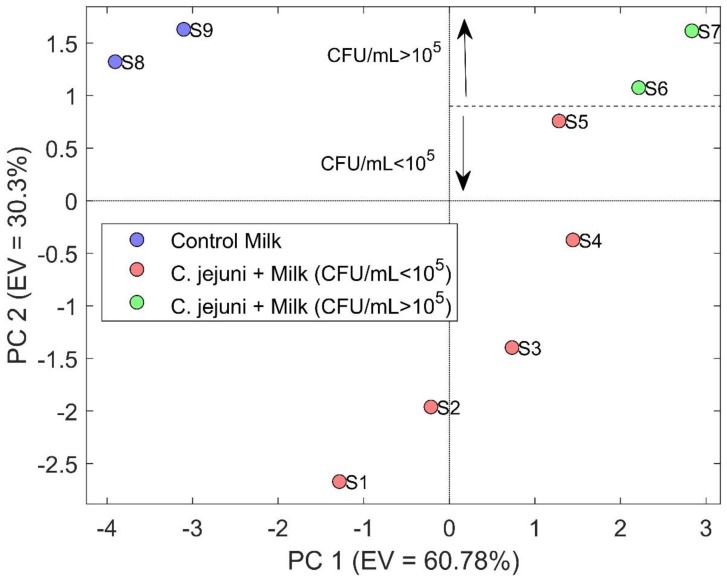
PCA score plot representing the data obtained during the first 7 h of the second set of analysis. The horizontal axis represents the explained variance enclosed in PC1 as equal to 60.78%, conversely, the vertical axis represents the explained variance enclosed in PC2 as equal to 30.30%. The total explained variance enclosed in the graphic is equal to 91.08%.

Regarding the sensor response, the obtained results are represented in Figure 3 as a form of PCA. The PCA score plot showed the first two principal components (PCs). The total explained variance (EV) was equal to 91.08% (60.78% for PC1 and 30.3% for PC2), meaning that most of the information was contained in two variables. A clear distinction between the contaminated samples and control milk was achieved considering the samples’ scores along PC1. 

Focusing attention on the contaminated specimens, the scores along PC2 can be assessed, which is helpful to understand the point where the threshold of 10^5^ is passed. Indeed, bearing in mind that a concentration of 1.5 × 10^4^ has been calculated from plate count at t = 0 h and *C. jejuni* doubles each 85 minutes [36], sample S5 contained 8.32 × 10^4^ CFU/mL, while sample S6 was 1.06 × 10^5^ CFU/mL, which oversteps the microbial acceptability for raw products that correspond to 10^5^ CFU/mL. In light of this result, the contaminated raw milk is not suitable for human consumption.

## 4. Conclusions

The obtained results pave the way for the S3 device to be applied in the dairy industry to prevent contaminations by *C. jejuni*. This paper illustrates the reaching of two different objectives, the first one was the differentiation between contaminated and not contaminated milk samples, and the second one was the follow up of the microbial development of the microbial specie and the limit detection of the milk salubrity. The obtained results completely fulfill the initial aim of the work. As future work, research will focus on the limit of detection of the instrument trying to reach the minimum detectable concentration in order to be able to actuate corrective actions before the milk reaches the not suitable for human consumption state. In addition, effort will concentrate into study investigating the effect of adding normal milk microflora to the samples in order to observe the interaction between the microorganisms and the volatiloma emitted from them. Experiments adding non-related microorganisms in order to demonstrate the specificity of detection is already on-going.

## Figures and Tables

**Table 1:** Type, composition, morphology, operating temperature, response (ΔR/R), selectivity (response ethanol/response carbon monoxide), and limit of detection (LOD) of ethanol for both S3 sensors arrays made at the SENSOR Laboratory.

Materials (Type)	Composition	Morphology	Operating Temperature (°C)	Response to 5 ppm of Ethanol	Selectivity	Limit of Detection (LOD) of Ethanol (ppm)
First Step Array						
SnO_2_Au (n)	SnO_2_ functionalized with Au clusters	RGTO	400 °C	6.5	3	0.5
SnO_2_ (n)	SnO_2_	RGTO	300 °C	3.5	2.5	1
SnO_2_ (n)	SnO_2_	RGTO	400 °C	4	2	0.8
SnO_2_Au+Au (n)	SnO_2_ grown with Au and functionalized with gold clusters	Nanowire	350 °C	7	2.5	0.5
SnO_2_Au (n)	SnO_2_ grown with Au	Nanowire	350 °C	5	2.1	1
CuO (p)	CuO	Nanowire	400 °C	1.5	1.5	1
**Second Step Array**						
SnO_2_Au (n)	SnO_2_ grown with Au	RGTO	400 °C	6.5	3	0.5
SnO_2_ (n)	SnO_2_	RGTO	400 °C	4	2	0.8
CuO (p)	CuO	Nanowire	350 °C	1.5	1.5	1
SnO_2_Au (n)	SnO_2_ grown with Au	Nanowire	350 °C	5	2.1	1
SnO_2_ (n)	SnO_2_	Nanowire	350 °C	4	2	0.8
